# Conceptualising and constructing ‘diversity’ through experiences of public and patient involvement in health research

**DOI:** 10.1186/s40900-021-00296-9

**Published:** 2021-07-22

**Authors:** Joanna Reynolds, Margaret Ogden, Ruth Beresford

**Affiliations:** 1 Department of Psychology, Sociology & Politics, Sheffield Hallam University, Heart of the Campus, Collegiate Crescent, Sheffield, S10 2BP UK; 2 Public Contributor, County Durham, UK

**Keywords:** Involvement, Patient, Public, PPI, Health research, Qualitative, Diversity, Experience

## Abstract

**Background:**

Increasing the accessibility of public and patient involvement (PPI) in health research for people from diverse backgrounds is important for ensuring all voices are heard and represented. Critiques of PPI being dominated by ‘the usual suspects’ reflect concerns over the barriers to involvement in PPI faced by people from minority groups or non-professional backgrounds. Yet, what has received less attention is how undertaking PPI work might produce diverse experiences, potentially shaping the motivation and capacity of people from different backgrounds to continue in PPI.

**Methods:**

We conducted qualitative research to explore experiences of the health research PPI field in the UK and to understand how these might shape the accessibility of PPI for people of diverse backgrounds. We conducted in-depth and follow-up interviews with five PPI contributors with experience of multiple health research projects, and a focus group with nine people in professional roles relating to PPI. Interview data were analysed using a narrative approach, and then combined with the focus group data for thematic analysis.

**Results:**

The structure, organisation and relationships of health research in the UK all shape PPI experiences in ways that can intersect the different backgrounds and identities of contributors, and can pose barriers to involvement and motivation for some. Navigating processes for claiming expenses can be frustrating particularly for people from lower-income backgrounds or with additional needs, and short-term research can undermine relationships of trust between contributors and professionals. Pressure on PPI coordinators to find ‘more diverse’ contributors can also undermine ongoing relationships with contributors, and how their inputs are valued.

**Conclusions:**

To increase diversity within PPI, and to ensure that people of different backgrounds are supported and motivated to continue in PPI, changes are needed in the wider health research infrastructure in the UK. More resources are required to support relationships of trust over time between contributors and professionals, and to ensure the unique circumstances of each contributor are accommodated within and across PPI roles. Finally, critical reflection on the pressure in PPI to seek ‘more diverse’ contributors is needed, to understand the impacts of this on those already involved.

## Plain English summary

Supporting people from different backgrounds to access public and patient involvement (PPI) roles in health research is important. Increasing ‘diversity’ in PPI is often thought about in terms of the demographic characteristics (eg gender, ethnicity) of people undertaking PPI roles. However, we also need to understand how people experience PPI differently, and what shapes their ability to become and continue being involved. We conducted in-depth and follow-up interviews with five experienced PPI contributors, and a focus group with nine professionals working in roles around PPI in health research in the UK, to explore their views and experiences of the PPI field over time.

Our findings show that how health research is organised impacts the motivation of people from different backgrounds to continue doing PPI, where processes for claiming expenses for PPI work can be particularly challenging for people on a low income and / or with additional needs. Relationships with PPI professionals are important for how valued different PPI contributors feel, but professionals’ expectations of PPI roles vary and some contributors feel their experience is not always welcomed. PPI coordinators can feel pressured by researchers to recruit new, ‘more diverse’ contributors to PPI roles, and they worry that this affects the relationships they have with people already doing PPI. Our research shows the need for more support within health research structures to enable people with different backgrounds and experiences to continue to feel valued and motivated in PPI. This will help ensure health research is informed by diverse perspectives.

## Background

With continuing recognition of the need to engage the public in the design and delivery of health research, public and patient involvement (PPI) is under increasing scrutiny in terms of how it is done, what impacts it has, and who is and who is not involved [1]. Amid critiques of the risk of PPI being tokenistic are broader questions about whose voices count within the health research space and whose views are being represented [2]. Linked to these concerns is the concept of ‘diversity’, and the drive to make PPI an inclusive space in which people from different backgrounds can participate equally [3] [4]. This focus on ‘diversity’ is typically framed around the demographic characteristics of contributors coming into PPI opportunities. However, less attention has been paid to how *doing* PPI work may be experienced in diverse ways, and how this might impact on different people’s capacity and motivation to pursue additional PPI roles, which ultimately shapes the diversity of the PPI field. In this paper we present findings from a qualitative study exploring how the organisation, structures and relationships of PPI work in the UK intersect the diverse circumstances and motivations of those involved. In doing so we seek to contribute to efforts to make PPI a more inclusive and supportive space.

### Diversity in PPI and why it is important

Increasing the diversity of people involved in PPI is important first from a democratic perspective, to ensure all groups are enabled to contribute and give their voice on the decisions that affect their lives [1, 3]. Second, from an inequalities perspective, the people who suffer the worst health and face more inequalities in accessing care are also those least likely to contribute to health research in a PPI capacity [5, 6]. When minority groups are marginalised from PPI structures, they miss out on opportunities to shape improvements to health care that would benefit their lives [5].

The emphasis on diversity can also be seen as a response to critiques that PPI work is dominated by the ‘usual suspects’ [7]. The ‘usual suspects’ typically come from more advantaged groups in society [8], usually white and of higher socio-economic status, often retired and from professional backgrounds, and thus have more capacity to become involved and articulate their views [2]. These concerns reflect debates around how representative PPI contributors are - or should be - of broader patient and public populations [9]. Not everyone will experience a health condition and services in the same way, and these experiences are likely to be shaped by aspects of their identity and inequalities they may already face. The emphasis on diversity also reflects wider social agendas around increasing inclusivity of organisations and systems in which minority groups are often under-represented, for example education and management [10, 11].

### Barriers to diversity within PPI

While the value of the patient / public perspective to health research is centred on lived experiences of a health condition or caring role [12], recent research has highlighted other skills often required for inputting successfully in health research spaces [2]. These include confidence with using technical language or articulating oneself with confidence in a formal meeting [12]. People who already have such capacities, for example those from professional backgrounds, may feel more comfortable undertaking PPI for health research, and those from other backgrounds may be marginalised or excluded [13]. Therefore, the lived experiences represented through PPI may be limited to people from a narrow set of backgrounds, and the views of a more diverse population, even with a similar health condition or caring role, may not be heard.

There is emerging evidence that the organisation of PPI in health research can exclude people from lower socio-economic backgrounds, ethnic minorities, and those with lower levels of literacy [14]. A recent review of the involvement of Black and Minority Ethnic (BAME) groups in health research highlighted multiple barriers in PPI practice that exclude these groups or make them feel uncomfortable contributing. These include lack of trust, challenges around communication and dismissal of cultural concerns, among others [6]. Other minority groups also face barriers to involvement including people lacking computer literacy, with different cognitive needs, without stable home addresses, and with mobility or communication difficulties [8, 15, 16]. It has been argued that failure to accommodate these issues and make PPI opportunities more accessible in a research study may reflect constraints on time or funding available for PPI activities [14]. However, it may also be indicative of a lack of understanding of the real experiences of different contributors within and across PPI roles, and the issues they encounter within the PPI field. With diversity and inclusivity recognised within established principles of good practice for PPI [17], more understanding is needed of how to support the different needs and interests of all those contributing, as well as how to make the PPI more accessible.

### Conceptualising diversity in relation to PPI

Much of the literature on increasing the diversity of people in PPI focuses on demographic categories such as gender, age, ethnicity and socio-economic status [6], with the presence or absence of people from sub-groups of these categories taken as a measure of diversity. This reflects what Hearn and Louvrier [10] describe as an ‘essentialist’ framing of diversity, focusing on aspects of individuals’ identities that are taken to be fixed and pre-existing. This is important for understanding who is and is not accessing PPI roles, but it tells us little about how people from different backgrounds and identities experience the PPI field more generally, and how motivated they are to continue to pursue PPI roles. Instead, we draw on Hearn and Louvrier’s ‘constructionist’ framing of diversity [10], to focus more on how differences between people are produced and experienced through the activities of PPI in health research. In this paper, we explore how the practices, organisation and structures of PPI shape the experiences of contributors, and thus influence the ‘diversity’ of perspectives shared in PPI spaces. In doing so, we seek to inform efforts to make PPI a more inclusive and democratic space, to influence health research and ultimately improve health and wellbeing for all.

## Methods

The focus of this paper draws from a UK-based qualitative study with the aim of exploring the experiences of PPI contributors over time, situated within the context of their broader lives. The study comprised in-depth and follow-up interviews with five experienced PPI contributors, and a focus group with nine people in professional roles connected to PPI in the UK. The results reported here draw from the focus group discussion and from how interview participants talked about their motivations, relationships and experiences in PPI. More detailed description of the in-depth interview methodology, and interview participants’ narratives of how PPI intersects *other* areas of their lives are reported elsewhere [18].

The focus group was conducted to explore the views of PPI professional stakeholders about changes in the PPI landscape in health research over time. Professional stakeholders were recruited to participate using existing PPI networks and contacts across the UK, and were eligible to participate if they held a professional role relating to PPI in health research (such as PPI coordinators / managers; academics and researchers interested in PPI; and people working in health research funding). Nine stakeholders participated in the focus group. The discussion was held in a meeting room at Sheffield Hallam University; six participants attended in person, and three participated virtually, via video conferencing. The focus group was audio recorded, lasted one hour and 45 min, and was facilitated by JR, with MO and RB acting as note-takers to record non-verbal communications. Participants were asked about their roles and experiences, their views on how PPI is valued in health research, relationships with PPI contributors, and ways to support and recognise PPI contributions.

In-depth and follow-up interviews were conducted with five experienced PPI contributors, recruited using social media and existing networks. People were eligible to participate if they had been involved in at least three health research projects in a PPI capacity, were over the age of 18, lived in the UK and were willing to be interviewed twice. Over 30 eligible people expressed interest, and five were chosen purposively to reflect a range of age, gender and types of PPI experience. Diversity of other characteristics (such as ethnicity, education level or socio-economic status) were not actively sought when recruiting interview participants, due to the sample size. However, participants’ varied backgrounds and identities emerged through their accounts of their experience; some of this is captured in the Findings below, and more detail is reported elsewhere [18]. Interviews were held at participants’ homes or at Sheffield Hallam University and were conducted by JR and RB. A narrative approach was taken [19], enabling participants to talk in an unstructured way about their experiences with PPI over time. Conducting follow-up interviews, between four and six weeks later, enabled a more in-depth exploration of their experiences [20]. Both the first and follow-up interviews were conducted by the same interviewer for continuity. The first round of interviews lasted an average of 85 min, and the second round, an average of 87 min, and all interviews were audio recorded.

All audio recordings were transcribed verbatim and participant names and other identifying details were anonymised; interview participants were invited to choose pseudonyms. The transcripts were supplemented by notes on non-verbal communication from the focus group, and reflective notes captured by the interviewers after each interview. The focus group transcript was analysed using an inductive thematic approach. First, JR and MO both conducted open coding of the data, working separately to identify ideas. Next, they compared their open coding and discussed their interpretations, before JR synthesised and refined the coding to eight themes. The interviews were first analysed by JR using a narrative approach to identify chronological stories of participants’ PPI experiences, and to explore how meaning was communicated through these stories; see [18] for more detail on the narrative analysis. Through discussion among all authors, the interview transcripts were then further analysed against the set of themes identified in the focus group transcript, enabling comparison between the perspectives of the professional stakeholders and PPI contributors.

## Results

Eight themes were identified through the analysis of the interview and focus group data, which were then organised into three analytical stories (see Table 1). These analytical stories reflect views on how PPI is organised and undertaken in different ways in UK health research, contributing to diversity of experience of PPI, including who is involved and how they feel about it. See Table 2 for a summary of the characteristics of the focus group and narrative interview participants. The insights are organised by analytical stories of how diversity of PPI experience is constructed 1) through health research infrastructure; 2) through the work of supporting PPI; and 3) in the expectations for and of PPI contributors.

**Table 1:** Summary of themes and analytical stories identified through the analysis

Theme	Analytical story
Research processes	1. Diversity of experience through research infrastructure
Support for PPI	2. Diversity of experience through supporting PPI
Relationships with PPI contributors	2. Diversity of experience through supporting PPI
Characteristics of PPI contributors	3. Diversity of experience through expectations for, and of, PPI contributors
Expectations for PPI	3. Diversity of experience through expectations for, and of, PPI contributors
Mechanisms of PPI	1. Diversity of experience through research infrastructure
Valuing PPI	1. Diversity of experience through research infrastructure
Motivations for doing PPI	3. Diversity of experience through expectations for, and of, PPI contributors

**Table 2:** Characteristics of participants

Focus group participants	Number (***n*** = 9)	Narrative interview participants^**a**^ (***n*** = 5)	Characteristics (gender, age range, years of PPI experience)
PPI coordinator / advisor / manager	6	Kendra	Female, 60s, > 15 years
PPI academic / researcher	2	Bhai	Male, 40s, 7 years
Funding organisation manager	1	Kat	Female, 60s, > 15 years
		Brendan	Male, 50s, 2 years
		Grace	Female, 50s, 20 years

^a^Interview participants are referred to by pseudonyms

### Diversity of experience through research infrastructure

The organisation of health research at an institutional, and even national level, shapes how PPI contributors and researchers experience and value PPI work, the kinds of knowledge contributors can offer, and how comfortable different contributors feel navigating the field. Across the focus group and interviews, there was recognition of the increasing range of opportunities for PPI across the research cycle, including in new contexts such as setting health research organisation strategies, or assessing the impact of research funding. PPI contributors also described undertaking multiple different PPI roles, and several contributors talked of actively seeking to expand their knowledge of the whole research process through PPI:

*“I took part in all sorts of things, you know, surgical trials, psychology studies, social science, palliative medicine. . . And then I thought . . . [I] would like to know about earlier in the process. So I applied for a funding panel and got on one; I've been on that for six years now.”* (Kat)

Focus group participants discussed the different kinds of knowledge and experience required from PPI contributors across this wide range of roles, including skills that are developed through continued engagement with research such as “*knowledge… of a methodology to comment on a particularly complex study”* (P06, PPI academic / researcher). Some PPI contributors echoed this view, talking of taking on certain PPI roles only when they had built up experience and understanding of research processes over time. This shows that increasing expectation for PPI across health research infrastructure leads to the development of varied sets of knowledge and experience among PPI contributors.

However, this may also result in tensions around how different kinds of knowledge and skills of contributors are valued by researchers, and what contributors feel they can offer. One interview participant, Kendra, narrated an experience of feeling uncomfortable in, and subsequently leaving, a PPI role when she realised that the researchers only wanted “*a raw input of experience*” from contributors. Kendra felt she was able to input into the research design, due to her experience in PPI: “*I was perhaps being too interfering with the research”.* This reflects debates on the tension between the ‘lay’ and ‘expert’ identities of PPI contributors [21]. Health research infrastructure can facilitate development of diverse knowledge and skills among PPI contributors, but this also may lead to conflicting expectations about what contributors can offer. As such, some contributors might feel their knowledge and experiences are unwelcome in certain PPI situations.

Our data also highlighted how there are different ways in which PPI work is valued, in terms of rewards or recompense offered to contributors, with potential implications for whose voices are perceived to ‘count’ in the PPI field. While a few focus group participants expressed doubt about the purpose of giving money to PPI contributors for certain tasks, others emphasised the ‘symbolic’ importance of paying PPI contributors to value their “*time and expertise*”. They also highlighted the value of non-monetary recognition, echoed by PPI contributors who appreciate being “*well looked after”*, for example being invited to conferences, helping them feel their work is valued. However, two interview participants also highlighted how the different structures for rewarding PPI contributions may discourage involvement. As a family carer with a low income, Bhai talked of the work involved in chasing up PPI payments and expenses across multiple institutions, often meaning he is ‘owed’ a considerable amount of money at any one time and affecting his choices around new PPI roles. Similarly, Grace described challenges she has faced in negotiating payment for expenses she incurs for PPI work due to additional needs related to a physical disability. Both Bhai and Grace’s narratives indicate potential barriers within the financial infrastructure of PPI work for people from particular economic situations and / or with additional needs.

Finally, the typical length of health researchers’ contracts in the UK can have impacts on how valued PPI contributors might feel, in different contexts. There was general agreement that good communication between researchers and contributors is important, including feeding back to PPI contributors at the end of a project on how their inputs have shaped the research. However, it was also recognised that this does not happen uniformly across projects, often because of the turnover of research staff due to short-term contracts. This can lead to a lack of continuity at the end of a project, resulting in experiences of frustration among contributors: “*the feedback [from researchers] is so poor*” (Grace). The way health research is organised in the UK means that PPI contributors face varied experiences in how they feel their knowledge is valued, financially and otherwise. This may contribute to different levels of (dis)comfort felt by contributors of varied backgrounds, potentially limiting the inclusiveness of the field.

### Diversity of experience through supporting PPI

The ways in which PPI work is supported within health research has potential to shape the relationships between researchers, PPI contributors and coordinators, and thus the ongoing access of contributors to roles within the field. Our research highlighted variability in how this support is organised, which in turn was perceived to influence how PPI work is seen to be valued. This could have implications for who does and does not feel motivated to continue pursuing PPI roles.

Focus group participants identified that the PPI coordinator role has become increasingly recognised as important in health research, “*to do good practice of PPI and avoid tick boxes*” (P09, PPI coordinator). However, participants identified a disparity of expectations and resources available for this role, across different institutions and research networks. They talked about the challenges they face around the motivation and capacity of researchers to engage with PPI activities, for example trying to ensure researchers share outputs with PPI contributors at the end of a project:

*“I do not have the time to follow up every single research project and say have you emailed these people [at the end of a project]?”* (P08, PPI coordinator).

Variation in how PPI support is resourced could therefore contribute to how PPI contributors experience the process differently, for example feeling undervalued when not receiving feedback on their input, as described above.

The focus group and interviews also highlighted different experiences of relationships between PPI contributors, coordinators and researchers. Several PPI contributors talked positively in their interviews about building up relationships with individual researchers over time, helping their learning of research processes and to feel valued. Kat described being “*very skilfully guided*” in the early stages of her PPI work by one researcher, across several projects, and talked of maintaining contact with another researcher who “*deals with us as equals*”. However, this closeness between certain PPI contributors and researchers can also be experienced as exclusionary. Grace raised concerns about processes for recruiting PPI contributors to new projects being biased and selective, stating that she feels researchers often have their “*pet patients and carers*”. She described turning down a PPI role in one study because she felt the opportunity had not been advertised to others equally, stating “*I can’t abide that kind of unfairness*”.

Focus group participants talked of different experiences of working with contributors, sometimes reflecting their varied professional experiences. PPI coordinators presented themselves as conduits between PPI contributors and researchers, but several indicated they have little opportunity to build connections with contributors aside from (usually) virtual contact to link them with PPI opportunities and events: “*it’s a very remote kind of way in which you’re handling people.”* (P07, PPI coordinator).

Participants discussed briefly the possible benefits of supporting researchers to ‘buddy up’ with PPI contributors, as helping to foster “sustainability” of PPI experiences, and to keep contributors connected and engaged with PPI work over the long term. This idea was echoed by Kendra who said it was important to her to have a sense of continuity between research projects, and that it can be “*really disappointing*” when a project ends without any further interaction.

Yet, the experience of one PPI academic / researcher highlighted how close working relationships between researchers and PPI contributors might be judged differently within the health research system. She recounted receiving criticism from research grant reviewers on the established collaboration between the research group and nominated PPI contributor:

“*We got reviewer comments back . . . on a grant where one of the co-applicants was a PPI partner who’d been involved in previous work with the group, and we were told we couldn’t have him as a co-applicant because he was too closely involved with the group and we needed somebody else. . . you would never say that to a research group about the statistician or the economist*” (P02, PPI academic / researcher).

Here, the research funding infrastructure reproduces different criteria against which PPI contributors and academic researchers are judged, which in turn shapes the ways in which PPI contributors find themselves positioned and valued. The variation in how relationships between contributors, researchers and coordinators are experienced within the health research system is potentially problematic. It may lead to disparity of access to support that enables and motivates PPI contributors to continue pursuing PPI roles, thus potentially restricting the diversity of knowledge that is available through the PPI function to inform health research.

### Diversity of experience through expectations for, and of, PPI contributors

How PPI contributors identify themselves and envisage their pathways through the PPI field varies greatly. Alongside this, there are expectations within the health research system for particular kinds of people to be brought into PPI roles. When these two sets of expectations are in tension, this may give rise to experiences of frustration and exclusion among contributors hoping to continue their PPI engagement.

Many focus group participants agreed that the term ‘diversity’ has become something of a “*buzzword that people are throwing around constantly*” (P08, PPI coordinator), and questioned how it is typically defined in relation to PPI, with one arguing “*it’s not really just.*. *. gender, culture*” (P05, PPI coordinator). Several PPI coordinators talked of the pressure they felt from researchers and research structures to find new and ‘diverse’ people for PPI roles, perceiving criticism if they are not able to bring in sufficiently ‘diverse’ people:

*“it’s sort of seen as a failing in me that the recruits that I have are all being, you know, what a researcher sees as the usual suspects”* (P07, PPI coordinator).

Focus group participants highlighted that the structure and timing of research activities, usually scheduled during the working day, may exclude people who work or have other responsibilities at these times, and there was a perceived reluctance among researchers to arrange meetings at other times. Moreover, despite the pressure for PPI coordinators to recruit people beyond the ‘usual suspects’ mould, some contributors described negative experiences of being of a minority identity within the health research context. Bhai told of sometimes being uncomfortable in research spaces, feeling looked down upon by senior researchers and clinicians due to his lack of comparable educational status, and possibly also his ethnicity: “*a second class citizen..*. *is it because I’m not white?*”. Here, the expectations for involving people from a diverse range of backgrounds was in tension with the current practical arrangements of PPI in health research, and the experiences of minority contributors within research spaces.

Focus group participants also discussed wider expectations to avoid involving people who have become ‘too professionalised’ within PPI, with the implication that they may have lost their perceived value as a ‘lay’ person. There were differences of opinion shared about what constitutes a ‘professionalised’ PPI contributor, and whether it is necessarily problematic. Among the contributors, a wide range of motivations were identified for getting involved in PPI initially, and for continuing in PPI; these are described in more detail elsewhere [18]. All contributors expressed a drive to pursue new PPI opportunities, and develop new skills and expertise, but for different reasons, including for social and financial status, and as a form of activism. For example, Brendan talked of how his motivation for PPI had shifted from staying active following a life-changing diagnosis to a way to disrupt hierarchies of decision-making around research and care for people with his condition:

*“I am always trying to build relationships with organisations and I am always challenging organisations and you know conferences where they don't have people with [condition] speaking. .. [and] I am moving more to challenging researchers when they don't involve people with [condition], being involved in developing research.”*

Focus group participants also recognised that PPI contributors have different motivations for pursuing PPI roles in health research and discussed the need for flexible options for getting new people involved in PPI work, whilst continuing to support those who wish to progress. This suggests that while attempting to meet the expectation for bringing (new) people from demographically diverse backgrounds into PPI roles, care must also be taken to recognise and support the varied motivations and needs of current PPI contributors.

These examples show how PPI is constructed and experienced in different ways across the health research system in the UK. These differences give rise to varied expectations of what the PPI role is or should be, and how it is valued and supported, which are not always aligned between contributors, coordinators and researchers. This may lead to frustrations, constraints and even exclusions of the contributions of certain groups of people, limiting the diversity of experience and knowledge represented in the PPI field, as demonstrated by the contributions of the participants in this research.

## Discussion

Concerns over the lack of diversity of people involved in PPI work in health research have emerged amid concerns over the representativeness of PPI contributors of the broader population [8], and persisting inequalities where those who face worse health outcomes are less likely to be involved in shaping health research [5]. Echoing diversity, equality and inclusion agendas in other fields, there has been particular focus on the demographic characteristics of the people coming *into* the PPI field, conceptualising diversity in terms of gender, ethnicity, age and socio-economic status. In this paper we sought to extend this work by looking at how diversity can be understood in relation to people’s experiences within and across PPI roles in the UK health research context. We drew on insights from in-depth and follow-up interviews with five experienced PPI contributors, and a focus group with nine professional stakeholders. We identified a range of structures, relationships and expectations around PPI work that lead to diverse experiences of PPI, and which shape the extent to which people from different backgrounds and identities feel comfortable and motivated to continue in the field. These findings have clear implications for efforts to increase the opportunities for diverse views and experience to help shape health research.

Expectations of undertaking PPI work and motivations for continuing with PPI are diverse among contributors, extending beyond health experiences, and evolving over time [18]. Recognising these different expectations, and finding ways to accommodate and support them, is vital for ensuring continuing inclusivity of opportunity in PPI. However, our research identified features of the health research infrastructure in the UK that potentially constrain this, including when PPI meetings and interactions occur; how PPI is supported and coordinated; relationships between PPI contributors, researchers and coordinators; and expectations for the PPI role. How these structures are differently experienced by PPI contributors, and how they intersect their personal situations and identities, all contribute to how accessible and supportive the PPI field is for different people. This builds on research by Jinks and colleagues [22] which highlighted the necessity of a health research system that can support the building up of trust and engagement with PPI contributors over time, to ensure contributions go beyond ad hoc, tokenistic inputs. Our research reveals the added implications of this for supporting the involvement of people from different backgrounds.

In highlighting the barriers to certain groups posed by the usual timing of PPI meetings, our findings support previous research emphasising the importance of making PPI opportunities more flexible and responsive to the needs of people from different backgrounds, and with different capacities to engage. Recommended approaches include using mobile PPI workshops to engage people with limited capacity to travel [7], or eschewing formal meetings for more informal ways of contributing [14]. Our research also emphasises the need for a longer-term perspective, recognising that health research infrastructure, such as institutional finance processes, may pose challenges to the continuing involvement of people with low income and / or additional needs. Parveen, Barker and colleagues [23] advocate a ‘person-centred approach’ to PPI, to accommodate individuals’ needs and preferences for PPI roles within a study. We suggest this approach should also be adopted across the wider research infrastructure, to ensure a diverse range of people are able to enter *and* continue to engage with PPI in ways that are supportive of their individual circumstances. Furthermore, other characteristics of the UK research infrastructure, such as the proliferation of short-term contracts for researchers, must be acknowledged as presenting barriers to effective relationships with PPI contributors, and therefore to their continued involvement.

Our findings also demonstrate the diversity of perspectives and experiences of those supporting PPI for health research in the UK. The role of the PPI coordinator (and similar positions) has been identified in recent research as “essential” for facilitating sustainable PPI relations between contributors and researchers ( [24]: p4). Yet, unlike the role of researchers (see for example [25], there has been surprisingly little research conducted into the coordinator role and its influence on experiences and outcomes of PPI in health research. Given the great potential for PPI coordinators to shape people’s PPI experiences, more attention to the diversity of their backgrounds and expectations is needed, to inform efforts to widen involvement in health research.

Furthermore, as our research shows, expectations of professional stakeholders towards PPI contributors can be in tension with the motivations and expectations of contributors themselves, and what knowledge they feel they can offer to research. This echoes debates around PPI contributors becoming ‘professionalised’ [26], reflecting concerns that contributors who become (too) experienced in research processes may lose the “grassroots credibility” attached to a particular health or caring identity ([12]: p612). However, as our research suggests, this assumption overlooks the multiple, evolving experiences and forms of knowledge that PPI contributors hold, which can be of value to research design. When coupled with a tokenistic diversity agenda that urges recruitment of people with particular demographic characteristics, this perspective risks neglecting the multiple identities that individuals have, and will develop through PPI work. When contributors’ expectations and needs are not met within the health research system, this may lead to reluctance to continue with PPI, and potential loss of a range of experiences and perspectives.

Finally, our findings suggest that the arguably tokenistic emphasis on ‘diversity’ within PPI systems may have negative consequences in terms of the expectations for PPI coordinators and researchers to find new, ever ‘more diverse’ contributors for their projects. The recent UK Standards for Public Involvement [17], which explicitly acknowledge the need for “inclusive opportunities” and for PPI roles to be “accessible”, are a good reminder of the importance of inclusivity in PPI. However, care must be taken around how these standards are used. The standards may discourage researchers from thinking critically about what specific PPI insight is relevant to their work [27] and inadvertently lead to the tokenistic, rather than meaningful, inclusion of people who meet particular demographic characteristics [8]. Furthermore, the formal emphasis on ‘diversity’ may undermine relationships between researchers, coordinators and established PPI contributors over time. While it is necessary to avoid PPI being dominated by a small group of people who cannot reflect the range of situations of people in the broader population [13], our research indicates the importance of continuity of relationships across projects. This can help PPI contributors to feel valued and motivated to continue, and those working with PPI to feel they can understand and support PPI contributors’ individual capacities appropriately, enabling meaningful – rather than tokenistic – contributions from a wide range of perspectives. This builds on recent research emphasising that establishing trust and collaboration through the development of relationships over time is vital for minority communities to feel confident in contributing to PPI work [7, 16, 23].

### Limitations

This study has several limitations which should be noted. First, the relatively small sample size of one focus group (of nine participants) and repeated narrative interviews with five PPI contributors limits the transferability of the findings to the wider communities involved in, and supporting PPI, in the UK and beyond. Although we recruited PPI contributors of different ages, gender and types of PPI experience, we did not actively seek to recruit people across different demographic categories such as ethnicity or socio-economic status given the small sample. However, the qualitative methodology chosen enabled a valuable level of depth of inquiry which revealed a diverse set of identities and experiences, even among the small sample. Coupled with the perspectives from across a range of PPI professional roles, this offers a good basis for further inquiry across a larger, and more mixed sample.

With three participants joining the focus group remotely, there were a few technological challenges which occasionally disrupted the flow of the discussion. The group nature of the discussion may have caused some participants to feel uncomfortable expressing alternative views in front of their peers, particularly around sensitive issues such as paying PPI contributors. However, care was taken by the facilitator to support participants to express different opinions, and this was reflected in multiple disagreements and (friendly) challenges among participants.

Finally, due to the networks used to recruit participants, the sample of professionals were mostly connected to health research funded by the largest funding bodies in the UK. Experiences and perspectives may vary among those doing and supporting PPI for health research funded by other organisations, and in contexts other than the UK. More comparative research is recommended to examine how diversity is constructed through PPI practices across different countries and health research infrastructures.

## Conclusions and recommendations

Exploring ‘diversity’ as something embedded in experiences of PPI work in health research, as well as a measurement of the demographic characteristics of contributors, helps to reveal the different ways in which the structures, relationships and expectations of PPI shape people’s motivations and capacities to pursue roles in the field. This is important for understanding not only who feels able to get involved with PPI in the first place, but also what enables them to continue applying their personal knowledge and expertise over time. A diversity agenda that (only) places emphasis on bringing new people into PPI roles risks being tokenistic and undermining the relationships and varied experiences of existing PPI contributors. As the field of PPI continues to evolve, for example with increasing use of virtual engagement arising from COVID-19 restrictions [28], renewed, critical attention is needed on how diverse experiences and perspectives are supported – and constrained – through the mechanisms of health research.

To ensure that PPI is inclusive of people from a wide range of backgrounds, and to ensure multiple and diverse experiences can continue to shape health research, changes to UK research infrastructure are required, beyond the production of checklists or other tools designed to increase ‘diversity’. PPI coordinators and similar roles must be well-resourced, to enable responsive relationships with contributors that facilitate involvement, and support individuals’ needs and interests over time. Efforts should be made to minimise loss of continuity resulting from researchers’ short-term contracts, and to improve systems for financial payments to PPI contributors, ensuring that individual requirements for undertaking PPI work are acknowledged and suitably recompensed. Although not directly discussed in this study, virtual mechanisms of engagement may offer increased accessibility to PPI for people who may otherwise struggle to attend meetings in person [29]. However, the potential for digital technology to exclude or deter certain groups poses additional issues around diversity of contributors, and must be considered carefully.

While efforts to recruit people from under-represented communities into PPI roles should continue, this should be accompanied by critical reflection on the research system’s tokenistic expectations for ‘diversity’, and how this may impact negatively on relationships with those already involved. Taking time to understand different backgrounds of those contributing to PPI is vital for building relationships of trust and value among all those involved in health research. This will help make PPI a more inviting and inclusive space for all, and in turn help ensure health research is impactful for those suffering the worst health.

## Data Availability

The datasets generated during the current study are not publicly available due to participants not consenting to this but are available from the corresponding author on reasonable request.

## Abbreviations

BAME: Black and Minority Ethnic
PPI: Public and patient involvement
